# *ETV6* germline mutations cause HDAC3/NCOR2 mislocalization and upregulation of interferon response genes

**DOI:** 10.1172/jci.insight.140332

**Published:** 2020-09-17

**Authors:** Marlie H. Fisher, Gregory D. Kirkpatrick, Brett Stevens, Courtney Jones, Michael Callaghan, Madhvi Rajpurkar, Joy Fulbright, Megan A. Cooper, Jesse Rowley, Christopher C. Porter, Arthur Gutierrez-Hartmann, Kenneth Jones, Craig Jordan, Eric M. Pietras, Jorge Di Paola

**Affiliations:** 1 Molecular Biology Graduate Program,; 2 Medical Scientist Training Program, and; 3 Division of Hematology, University of Colorado Anschutz Medical Campus, Aurora, Colorado, USA.; 4 Department of Pediatrics, Children’s Hospital of Michigan, Wayne State University, Detroit, Michigan, USA.; 5 Department of Pediatrics, Children’s Mercy Hospital, Kansas City, Missouri, USA.; 6 Department of Pediatrics, Washington University at St. Louis, St. Louis, Missouri, USA.; 7 Department of Internal Medicine, University of Utah, Salt Lake City, Utah, USA.; 8 Department of Pediatrics, Emory University School of Medicine, Atlanta, Georgia, USA.; 9 Department of Internal Medicine and; 10 Department of Biochemistry and Molecular Genetics, University of Colorado Anschutz Medical Campus, Aurora, Colorado, USA.; 11 Department of Cell Biology, University of Oklahoma Health Sciences Center, Oklahoma City, Oklahoma, USA.

**Keywords:** Hematology, Transcription

## Abstract

ETV6 is an ETS family transcription factor that plays a key role in hematopoiesis and megakaryocyte development. Our group and others have identified germline mutations in *ETV6* resulting in autosomal dominant thrombocytopenia and predisposition to malignancy; however, molecular mechanisms defining the role of *ETV6* in megakaryocyte development have not been well established. Using a combination of molecular, biochemical, and sequencing approaches in patient-derived PBMCs, we demonstrate abnormal cytoplasmic localization of ETV6 and the HDAC3/NCOR2 repressor complex that led to overexpression of HDAC3-regulated interferon response genes. This transcriptional dysregulation was also reflected in patient-derived platelet transcripts and drove aberrant proplatelet formation in megakaryocytes. Our results suggest that aberrant transcription may predispose patients with *ETV6* mutations to bone marrow inflammation, dysplasia, and megakaryocyte dysfunction.

## Abstract 2

Germline mutations in transcription factor ETV6 cause cytoplasmic localization of the HDAC3/NCOR2 complex, substantively altering the transcriptional signature of blood cells.

## Graphical abstract

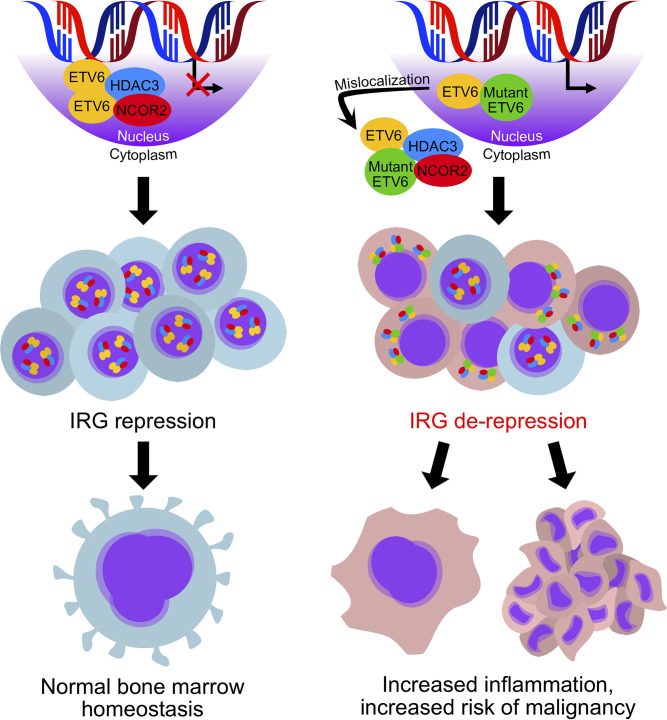

## Introduction

ETV6, the E26 transformation-specific (ETS) family transcription repressor and tumor suppressor variant 6 gene, resides on chromosome 12p13 and encodes a 57 kDa ETS family transcription factor with 3 highly conserved functional domains (1, 2): an N-terminal pointed (PNT) dimerization domain, a central linker domain, and an 85–amino acid C-terminal ETS DNA-binding domain, which associates with the 5′GGA(A/T)3′ motif in the promoter region of target genes (2). The ETS family of transcription factors represents a vast network of inter- and intramolecular interactions that result in combinatorial regulation of gene expression (2–7). Dimerization of ETV6 through the PNT domain facilitates cooperative DNA binding and transcriptional regulatory activity (8). While ETV6 is known to dimerize to achieve its transcriptional function, it has been reported to associate with other corepressors, such as HDAC3, NCOR, and Sin3 (9). Indeed, molecular models have defined ETV6 as a transcriptional repressor (9–12), likely via its interaction with HDAC-related corepressors (9, 12, 13).

ETV6 is a well-studied hematopoietic transcription factor previously reported to play a key functional role in hematopoiesis, megakaryopoiesis, cell growth, and differentiation (11, 14–16). Previous studies in mouse models indicate that ETV6 is necessary for normal hematopoiesis (14, 16, 17). As such, global deletion of *Etv6* in mice results in embryonic lethality between E10.5 and E11.5, with yolk sac angiogenic defects (16, 17). Conditional knockout of *Etv6* in megakaryocyte (MK) erythroid progenitor cells in mice results in thrombocytopenia, with an increased frequency of MK colony-forming cells (14). These findings are consistent with a terminal defect in MK maturation, leading to a compensatory increase in MK progenitor cells. *Etv6* controls the survival of hematopoietic stem cells (HSCs) and is required late in the development of MKs, where it may be acting in concert with other transcriptional regulators of megakaryopoiesis to bind MK-specific promoters (14). In human studies, ETV6 has been shown to regulate the expression of platelet and MK-specific proteins, such as MK/platelet glycoprotein 1bα (*GP1BA*) and glycoprotein IX (*GP9*) (18, 19). Furthermore, ETV6 interacts with FLI1 (18), another ETS transcription factor and master regulator of MK development. FLI1 haploinsufficiency results in Paris-Trousseau syndrome, another heritable form of thrombocytopenia (20). Thus, ETV6 is known to have a critical role in MK development and platelet production.

We and others have identified heterozygous germline *ETV6* mutations as the cause for dominantly inherited thrombocytopenia in several families (11, 15). The majority of the mutations reported to date occur in the ETS DNA-binding domain, where they have been shown to decrease DNA-binding capacity (12). The p.Pro214Leu mutation in the central domain is the most commonly identified mutation outside of the ETS domain, occurring in 5 unique families (21). Deletions in the ETS domain have also been described, resulting in protein truncation as a consequence of alternative splicing (22, 23). In cell lines and overexpression models, missense mutations in the central domain and the ETS DNA-binding domain of ETV6 result in aberrant cytoplasmic localization, decreased transcriptional repression, and impaired MK maturation (11, 15). The bone marrow of these affected individuals shows erythroid dysplasia and hyperplasia of small, hypolobulated, immature MKs, suggesting incomplete differentiation and inability to release platelets into circulation (11). Despite these recent advancements in the field, the precise molecular mechanisms underpinning thrombocytopenia and cancer predisposition in patients with *ETV6* mutations have yet to be defined.

In this study, we sought to define the role of ETV6 in human blood cells and understand the cellular and transcriptional consequences of *ETV6* germline mutations. We used PBMCs from healthy controls and patients carrying germline mutations in the central domain (p.ProP214Leu; P214L) and the ETS DNA-binding domain (p.Arg369Gln; R369Q) to investigate the subcellular localization, protein-protein interactions, and transcriptional targets of ETV6. Here, we demonstrate abnormal subcellular distribution of ETV6 and the HDAC3/NCOR2 repressor complex. This cytoplasmic mislocalization leads to upregulation of proinflammatory interferon response genes, which are regulated by HDAC3 (24). We also interrogated the consequence of cytoplasmic ETV6 in MK development and demonstrated aberrantly upregulated proinflammatory transcripts in platelet mRNA expression profiles from patients carrying the ETV6 P214L allele. We further confirmed this transcriptional signature by ablating nuclear ETV6 in MKs and show increased proinflammatory interferon response gene expression and significantly reduced proplatelet formation. Taken together, these data demonstrate that *ETV6* is required for homeostatic MK programing and differentiation through its association with HDAC3/NCOR2. Nuclear exclusion of ETV6 shifts the cellular balance of histone and protein acetylation, altering the epigenetic landscape and derepressing interferon response genes. Aberrant gene expression in MKs results in defective proplatelet formation, underpinning the thrombocytopenia observed in patients with germline mutations in *ETV6*.

## Results

### Germline ETV6 mutations cause cytoplasmic localization of ETV6 in all nucleated blood cells.

**Figure 1:**
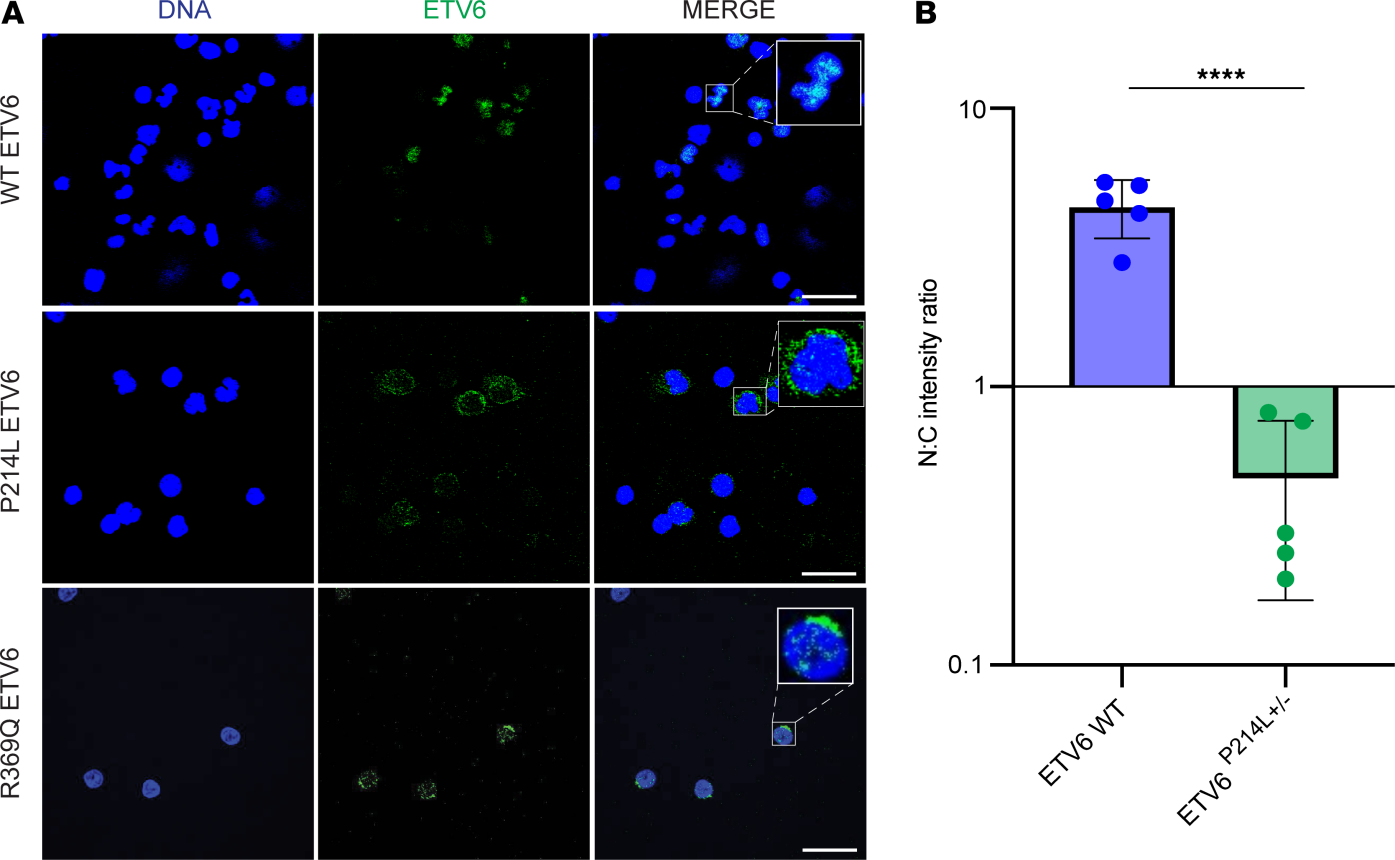
Mislocalization of ETV6 P214L in all peripheral cell types. (**A**) Comparison via immunofluorescence microscopy of ETV6 localization in PBMCs from ETV6 P214L and R369Q patients and healthy controls. Representative PBMCS stained for DNA (blue) and ETV6 (green). In cells from healthy controls, ETV6 is concentrated in the nucleus. In contrast, cells from patients carrying ETV6 P214L and ETV6 R369Q demonstrated ETV6 concentrated in the cytoplasm, with scarce staining in the nucleus. Scale bar: 20 μm. Original magnification, ×8 (inset). (**B**) Nuclear-to-cytoplasmic intensity ratio of ETV6 is significantly reduced in ETV6 P214L PBMCs (unpaired *t* test, ****P < 0.0001). Fluorescence intensity was calculated in the nucleus and cytoplasm. *n* = 5 random fields of view used to calculate N/C intensity ratio, representing over 100 cells analyzed in each condition.

While mislocalization of mutant ETV6 has been reported in overexpression models and cell lines (11, 15), this phenotype has not been validated in patient-derived cells expressing physiologic amounts of ETV6. We performed immunofluorescence studies to examine the subcellular localization of ETV6 in PBMCs derived from healthy controls and patients carrying germline mutations in *ETV6*. In healthy control PBMCs, ETV6 was concentrated in the nucleus, colocalizing with DNA (Figure 1A). However, in patients carrying the germline mutations P214L and R369Q, ETV6 was concentrated in the cytoplasm (Figure 1A). Quantification of ETV6 fluorescence intensity in each subcellular compartment demonstrated a significant alteration of the nuclear-to-cytoplasmic ratio of ETV6 P214L (Figure 1B, unpaired *t* test, *P* < 0.0001). The statistical analysis of R369Q samples was limited due to low cell numbers; however, the staining pattern was conserved. Mislocalized ETV6 was observed across all patient samples analyzed (*n* = 3 patients carrying ETV6 P214L [ETV6 P214L patients] and patients carrying ETV6 R369Q mutations [ETV6 R369Q patients], respectively). These data indicate that mutant ETV6 is mislocalized to the cytoplasm in patient-derived PBMCs, in accordance with previous studies using overexpression tools and cell lines.

### ETV6 associates with HDAC3 and mislocalizes the HDAC3/NCOR2 complex, resulting in increased histone acetylation.

**Figure 2:**
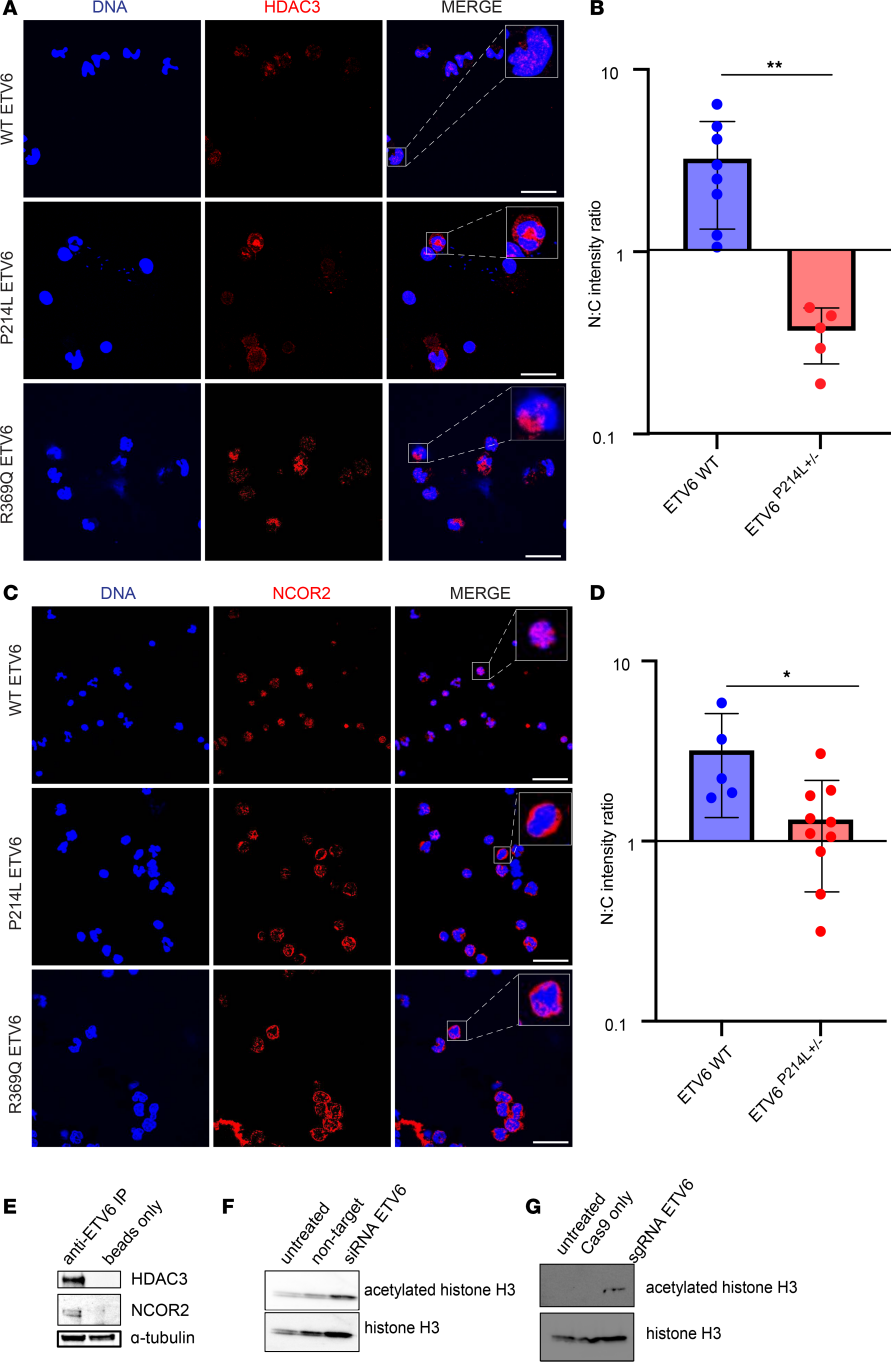
HDAC3/NCOR2 complex binds to ETV6 and is mislocalized in ETV6 P214L and R369Q PBMC, resulting in increased acetylation. (**A**) Comparison via immunofluorescence microscopy of HDAC3 localization in PBMCs from ETV6 P214L and R369Q patients and healthy controls. Representative PBMCs stained for DNA (blue) and HDAC3 (red). In cells from healthy controls, HDAC3 is concentrated in the nucleus. In contrast, cells from patients carrying ETV6 P214L or R369Q demonstrated HDAC3 concentrated in the cytoplasm, with scarce staining in the nucleus. Scale bar: 20 μm. Original magnification, ×8 (inset). (**B**) Nuclear-to-cytoplasmic intensity ratio of HDAC3 is significantly reduced in ETV6 P214L PBMCs (unpaired *t* test, **P = 0.0071). Fluorescence intensity was calculated in the nucleus and cytoplasm. *n* = 5 random fields of view were used to calculate N/C intensity ratio, representing a minimum of 100 cells analyzed in each condition. (**C**) Comparison via immunofluorescence microscopy of NCOR2 localization in PBMCs from ETV6 P214L patients and healthy controls. Representative PBMCs stained for DNA (blue) and cofactors listed above (red). In cells from healthy controls, NCOR2 is concentrated in the nucleus. In contrast, cells from patients carrying ETV6 P214L and R369Q demonstrated NCOR2 concentrated in the cytoplasm, with scarce staining in the nucleus. Scale bar: 20 μm. Original magnification, ×8 (inset). (**D**) Nuclear-to-cytoplasmic intensity ratio of NCOR2 is significantly reduced in ETV6 P214L PBMCs (unpaired *t* test, *P = 0.0169). Fluorescence intensity was calculated in the nucleus and cytoplasm. *n* = 5 random fields of view were used to calculate N/C intensity ratio, representing a minimum of 100 cells analyzed in each condition. (**E**) Immunoprecipitation of ETV6 in healthy control PBMCs demonstrates ETV6 interacting with HDAC3 and NCOR2. α-Tubulin was included to demonstrate equal loading of protein in each sample. Representative image of 2 independent experiments. (**F**) Transient knockdown of *ETV6* by siRNA in healthy control PBMCs results in increased acetylated histone H3 compared with control conditions. Representative image of 2 independent experiments. (**G**) CRISPR-mediated knockout of *ETV6* in cord blood–derived CD34^+^ HSCs drives increased acetylation of histone H3. Representative image of 2 independent experiments.

While the ETV6 PNT and ETS domains have been defined as the dimerization and DNA-binding regions, respectively (1, 10), the function of the central domain of ETV6 has been largely unexplored. Previous reports in overexpression models have shown interaction between the central domain of ETV6 and transcriptional corepressors, such as HDAC3 and NCOR (9, 13). These components of the ETV6 transcriptional machinery have been proposed to act as corepressors of ETV6 transcriptional targets (13). With our observation of cytoplasmic ETV6 in patient-derived PBMCs, we hypothesized that cofactors we identified in a complex with ETV6 would also be mislocalized to the cytoplasm. Thus, we demonstrate by immunofluorescence staining of HDAC3 (Figure 2A) a significant mislocalization to the cytoplasm mutant ETV6 in ETV6 P214L cells. Quantification of HDAC3 fluorescence intensity in each subcellular compartment demonstrated a significant alteration of the nuclear-to-cytoplasmic ratio (Figure 2B, unpaired *t* test, *P* = 0.0071). We further demonstrated altered subcellular localization when examining NCOR2 in the patient-derived PBMCs (Figure 2C). Quantification of fluorescence intensity of NCOR2 in each subcellular compartment demonstrated a significant alteration of the nuclear-to-cytoplasmic ratio of NCOR2 in ETV6 P214L cells (Figure 2D, unpaired *t* test, 0.0169). We validated the biochemical interaction of this complex in primary PBMCs derived from healthy controls and showed by coimmunoprecipitation a robust protein-protein interaction among ETV6, HDAC3, and NCOR2 in PBMCs expressing WT ETV6 (Figure 2E; see complete unedited blots in the supplemental material). Previous studies using a two-hybrid approach demonstrate both WT and ETV6 P214L to associate with these nuclear corepressors (12), further confirming our observation of this extranuclear transcriptional complex in patient-derived PBMCs.

We next hypothesized that cytoplasmic mislocalization of the ETV6/HDAC3/NCOR2 transcriptional complex in PBMCs would result in increased histone acetylation due to loss of nuclear HDAC3 activity. To recapitulate the lack of nuclear ETV6 we observed in the patient-derived PBMCs, we significantly reduced the expression of ETV6 in healthy control primary PBMCs using an siRNA-mediated knockdown of ETV6 (*P* < 0.0001, Supplemental Figure 3A). We also ablated ETV6 expression in primary cord blood–derived MKs using CRISPR-mediated deletion of *ETV6*. We observed increased acetylation of histone H3 using both strategies in these unique primary cell types (Figure 2, F and G; see complete unedited blots in the supplemental material). The altered balance of acetylated histone H3 in the absence of nuclear ETV6 indicates epigenetic changes driving transcriptional dysregulation in PBMCs.

### Cytoplasmic localization of ETV6 in P214L and R369Q patient–derived PBMCs drives upregulation of interferon response genes.

**Figure 3:**
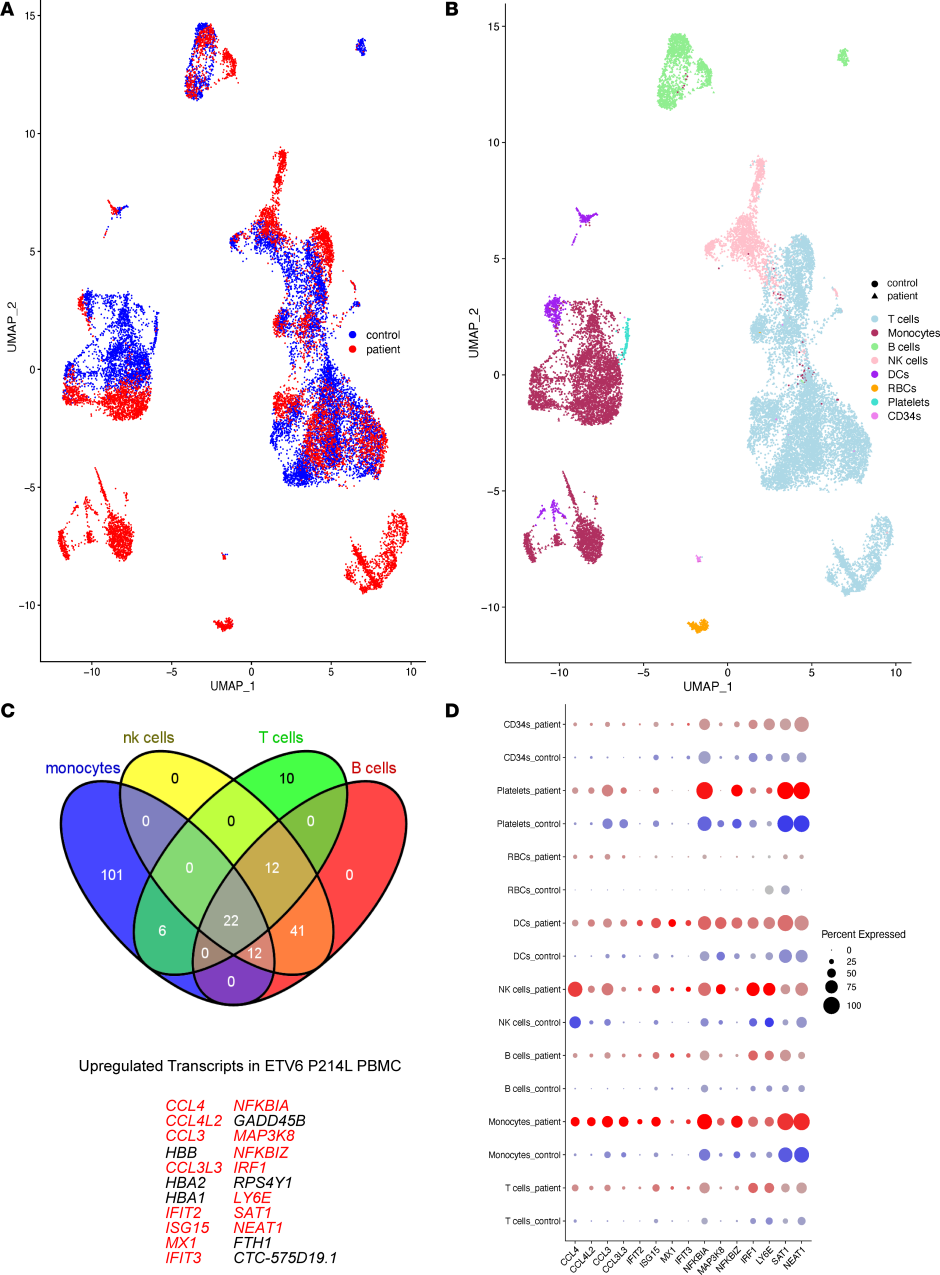
Cytoplasmic localization of ETV6 in P214L and R369Q patient-derived PBMCs drives upregulation of interferon response genes. (**A**) UMAP plot of cells sequenced from healthy controls (blue) and ETV6 P214L patients (red). (**B**) UMAP plot of cells sequenced from healthy controls (circles) and ETV6 P214L patients (triangles), denoting cell types. (**C**) Complex Venn diagram of upregulated genes across all 4 cell subsets, identifying a highly specific 22 gene signature of proinflammatory interferon response genes (in red). (**D**) Dot plot of top differentially expressed interferon response genes, split by ETV6 P214L patients versus age and sex matched controls.

We observed aberrant subcellular localization of ETV6 and the HDAC3/NCOR2 complex in all PBMCs from patients with the P214L and R369Q mutations and increased histone acetylation when ETV6 expression was reduced in the nucleus in healthy PBMCs and cord blood–derived MKs. To determine the transcriptional consequence of nuclear exclusion of ETV6, we performed single-cell RNA-Seq of PBMCs from affected individuals carrying ETV6 P214L (*n* = 5 patients) and R369Q (*n* = 3 patients) mutations and compared their gene expression with healthy age- and sex-matched controls (*n* = 6 donors and 3 donors, respectively) (Figure 3A). Using well-defined surface markers and transcriptional signatures, we identified broad peripheral cell populations, such as B cells (*MS4A1*, *CD19*), T cells (*IL7R, CCR7, S100A4*), NK cells (*GNLY, NKG7*), and monocytes (*CD14, LYZ, MS4A7*), on the Uniform Manifold Approximation and Projection (UMAP) projection of the cell clusters (Figure 3B) (25). We then used this data set to investigate transcriptional differences between patients and controls, and we detected upregulation of 204 genes representing a highly robust proinflammatory transcriptional signature. We analyzed the intersection of the increased transcripts across these 4 populations (Figure 3C), which identified a highly specific suite of 22 genes that were upregulated 4- to 400-fold depending on the cell type. These highly upregulated 22 transcripts in the affected individuals were highly enriched for the interferon response pathway, consisting of proinflammatory transcripts, such as *CCL4*, *CCL4L2*, *CCL3*, *CCL3L3*, *IFIT2*, *ISG15*, *MX1*, *IFIT3*, *IRF1*, and *NFKBIA*, among others (Figure 3D). PBMC transcripts from ETV6 R369Q patients confirmed this result, with interferon response genes being upregulated in patient samples when compared with control cells (Supplemental Figure 1; supplemental material available online with this article; https://doi.org/10.1172/jci.insight.140332DS1). Supporting our initial observations of ETV6 interaction with the HDAC3/NCOR2 complex, HDAC3 was confidently predicted by Ingenuity Pathway Analysis to be an upstream master regulator of this suite of upregulated genes (Supplemental Figure 2). These observations suggest that ETV6 and HDAC3 coordinately regulate the expression of interferon response genes.

To further confirm this observation, we used our siRNA knockdown model in healthy control PBMCs. Upon robust protein and mRNA knockdown of ETV6 (Supplemental Figure 3A, Mann-Whitney *U* test, *P* > 0.0001), we subjected the PBMCs to RNA-Seq and performed differentially expressed gene (DEG) analysis on the control (untreated and nontargeting) and ETV6 knockdown samples (Supplemental Figure 3B, 3 independent experiments). From this DEG analysis, we observed upregulation of proinflammatory genes, such as *CCL3*, *CCL7*, *IRF1*, *IRF4*, *IFIT2*, and *IL1B*, among others when comparing our control samples to ETV6 knockdown samples. These results were not statistically significant due to the variability of ETV6 knockdown in primary cells between independent experiments. However, top canonical pathways identified by Ingenuity Pathway Analysis of these DEGs demonstrated upregulated neutrophil/monocyte inflammation (TREM1 signaling, ref. 26), regulation of macrophage inflammatory signaling (LXR/RXR activation, ref. 27, 28; iNOS signaling, ref. 29), and inflammatory cytokine signaling (IL-6 signaling, role of IL17-F in allergic inflammation, ref. 30) (Supplemental Figure 3C). Taken together, these data indicate that nuclear ETV6 modulates the repression of key inflammatory transcripts.

To control for frequency of PBMCs and eliminate skewing of transcriptional changes due to cell numbers, we used flow cytometry–based immunophenotyping to compare mutant ETV6 PBMCs to healthy controls. Immunophenotyping of both ETV6 P214L and ETV6 R369Q PBMCs demonstrated normal frequencies of peripheral cell populations, including B and T cells (CD3^+^ and CD19^+^, respectively) and common lymphoid progenitors (CD19^–^CD3^–^CD34^+^CD10^+^) (Supplemental Figure 4). Of note, the abundance of peripheral circulating HSCs (CD34^+^, CD38^–^, CD90^+^) was significantly increased in patients carrying the ETV6 P214L allele (Supplemental Figure 4A), which confirms previous observations of increased circulating HSCs in other families carrying this mutation (12). ETV6 R369Q patients also had increased peripheral MK erythroid progenitors (CD10^–^CD34^+^CD38^+^CD135^–^CD45RA^–^) (Supplemental Figure 4B). To assess whether the inflammatory gene signature in ETV6 P214L PBMCs was associated with systemic inflammatory cytokine production, we analyzed the cytokine profile of the ETV6 P214L patients as compared with that of healthy controls. However, we did not observe increased proinflammatory cytokines or chemokines in ETV6 P214L patients, suggesting that the transcriptional differences observed between the genotypes are a result of mutated ETV6 and not due to another ongoing proinflammatory processes (Supplemental Figure 5). They also suggest the inflammatory gene signature may not directly translate to increased systemic cytokine production in ETV6 P214L patients.

### Patient-derived ETV6 P214L platelets and MKs lacking nuclear ETV6 show upregulation of interferon response genes and decreased proplatelet formation.

**Figure 4:**
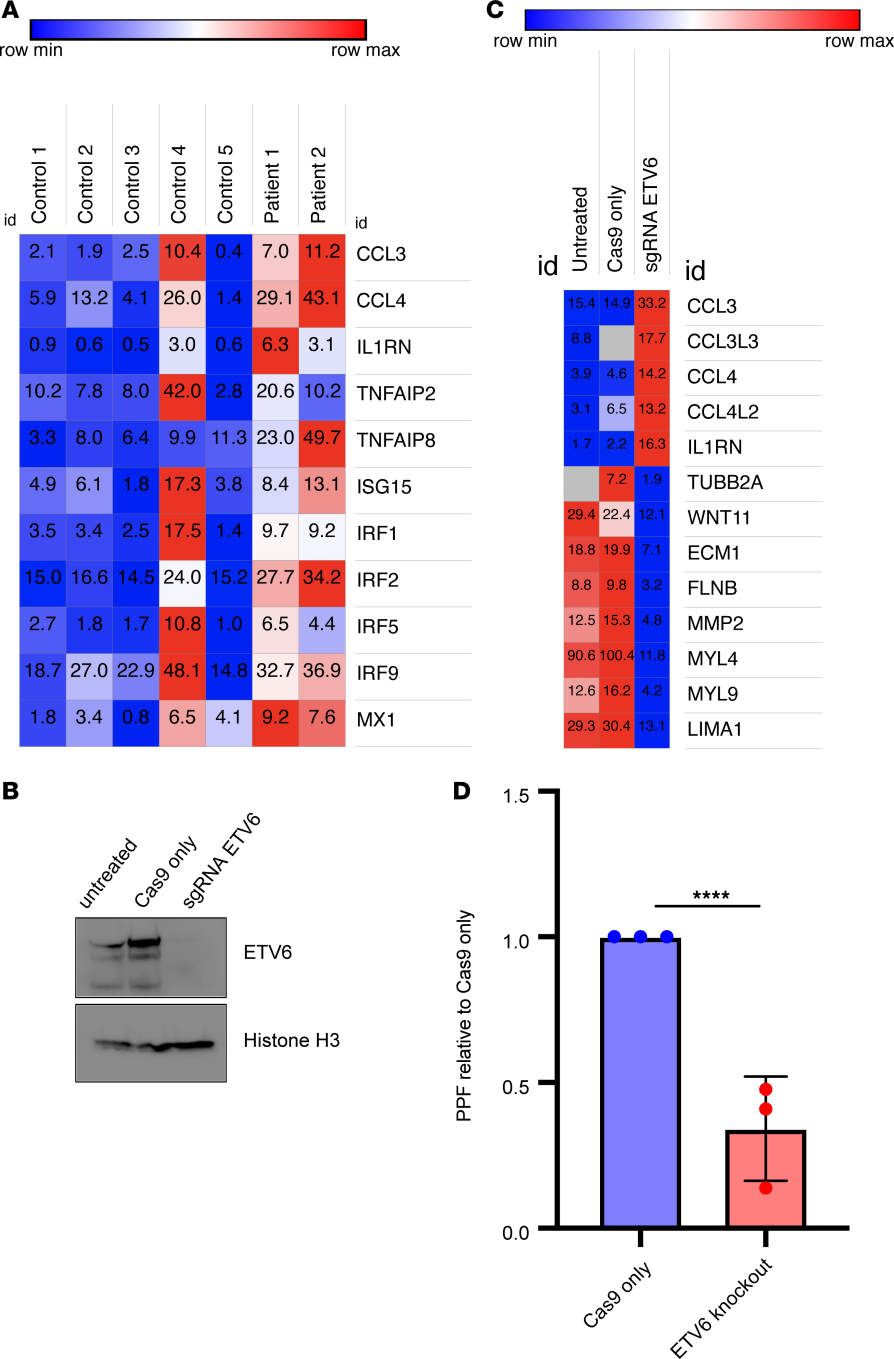
ETV6 P214L–derived platelets and MKs lacking nuclear ETV6 demonstrate transcriptional dysregulation and impaired proplatelet formation. (**A**) Proinflammatory interferon response genes are dysregulated in platelet transcripts from ETV6 P214L patients (*n* = 2) as compared with healthy controls (*n* = 5) (adjusted *P* > 0.0001 for all genes displayed). (**B**) Western blot demonstrating complete knockout of ETV6 protein mediated by CRISPR sgRNA. Representative of 2 independent experiments. Histone H3 loading control is previously shown in Figure 2G, as these experiments were conducted on the same set of knockout samples. (**C**) Transcriptional dysregulation of ETV6-knockout MK cells, upregulated genes enriched in the interferon response pathway, downregulated genes enriched in cytoskeletal biology. Heatmap displays mean transcript counts of 1 experiment in a series of 3 independent experiments. (**D**) Proplatelet formation is significantly impaired (Mann-Whitney *U* test, *P* < 0.0001, 3 independent experiments) when ETV6 is deleted in cord blood–derived MKs. CD34^+^ cells were differentiated into MKs, and proplatelet-forming cells were scored on day 12–14. More than 300 proplatelet-forming cells were counted per condition.

Despite the well-defined association between germline mutations in *ETV6* and thrombocytopenia, the transcriptional role of ETV6 in human MK maturation and platelet production is still unknown. We hypothesized that patient-derived platelets would exhibit upregulation of interferon response genes, as ETV6 is a ubiquitously expressed transcriptional repressor in hematopoietic cells. Furthermore, we sought to understand the transcriptome-wide alterations caused by cytoplasmic mislocalization of ETV6 during MK development. We compared the profile of platelet mRNA derived from patients carrying the ETV6 P214L allele (*n* = 2) to that of healthy controls (*n* = 5, 2 unaffected relatives and 3 unrelated) and observed upregulation of key interferon response genes, such as *CCL3*, *CCL4*, *IL1RN*, *TNFAIP2/8*, *ISG15*, *IRF1/2/5/9*, and *MX1* (Figure 4A, adjusted *P* < 0.0001 for all genes displayed). Additionally, we observed 2 clusters of downregulated transcripts that were identified by DAVID Functional Annotation Clustering (Supplemental Figure 6). The most enriched cluster included extracellular matrix and cell adhesion transcripts (enrichment score = 1.8), and the second-most enriched cluster included genes involved in cytoskeletal biology (enrichment score = 1.78). The differences we observed in the platelet transcriptome of affected patients suggest that *ETV6* plays a key role in megakaryopoiesis; however, the platelet transcriptome likely represents a snapshot of the more complex MK transcriptional landscape. Thus, we confirmed the transcriptional signature we observed in the patient-derived platelet transcripts in MKs by ablating *ETV6* using CRISPR/Cas9 gene editing. We achieved ETV6 deletion in primary CD34^+^ cells derived from umbilical cord blood and differentiated these cells into MKs as previously reported (11, 31) (Figure 4B; see complete unedited blots in the supplemental material). We compared the transcriptional profile of cells expressing ETV6 to those lacking ETV6 (Figure 4C), and, similar to that in patient-derived platelets and PBMCs, we found upregulation of proinflammatory transcripts, including key interferon response genes, such as *CCL3*, *CCL3L3*, *CCL4*, *CCL4L2*, and *ILRN* in ETV6-deficient MKs (Figure 4C, representative heatmap of 1 experiment in a series of 3 independent experiments). Notably, we observed downregulation of several cytoskeletal transcripts, such as *TUBB2A*, *WNT11*, *ECM1*, *FLNB*, *MMP2*, *MYL4*/9*,* and *LIMA1*, as was initially observed in the patient-derived platelets (Figure 4C). We further observed significantly decreased proplatelet formation in the ETV6-knockout MKs (Figure 4D, 3 independent experiments, Mann-Whitney *U* test, *P* > 0.0001), demonstrating that ETV6 is required for functional megakaryopoiesis and platelet formation. Consistent with clinical findings in patients carrying germline ETV6 mutations (11, 12), MKs that lack nuclear ETV6 are similarly deficient in proplatelet formation. Collectively, these data suggest that ETV6 is required for homeostatic transcriptional control in MKs, and the altered interferon and cytoskeletal gene programs we observed may predispose patients with ETV6 mutations to thrombocytopenia via decreased proplatelet formation.

## Discussion

Here we describe 2 *ETV6* germline mutations residing in distinct domains of the protein that causes cytoplasmic mislocalization not only of ETV6, but also of the ETV6-associated HDAC3/NCOR2 complex, altering the transcriptional profile of PBMCs and impairing MK proplatelet formation. The function of ETV6 has primarily been investigated with the use of overexpression models, reporter plasmids, and cell lines. While these tools have been valuable in characterizing the structure and protein interactions of ETV6, the transcriptional targets and physiologic role of ETV6 had not yet been shown in patients.

Given our findings that ETV6 is mislocalized from the nucleus of patient-derived PBMCs, we sought to define the transcriptome of patients with germline ETV6 mutations. We demonstrated that ETV6 P214L and R369Q PBMCs had upregulated proinflammatory interferon response genes, which are known to be regulated by HDAC3 (24). We further showed that knockdown of ETV6 in primary PBMCs and ablation of ETV6 in primary cord blood–derived MKs skews the balance of acetylated histone H3. While these effects are likely cell-type specific, the increase in histone acetylation observed as a result of ETV6 knockdown and/or ablation demonstrates the functional consequence of cytoplasmic ETV6, which is likely secondary to the inability of HDAC3/NCOR2 to access nuclear targets. These findings suggest that nuclear ETV6 is required to direct the HDAC3/NCOR2 complex to interferon response promoters, and the lack of deacetylation of these specific promoters, as seen by increased acetylated histone H3, is sufficient to derepress their transcription. Importantly, patient-derived platelet transcripts demonstrate upregulation of interferon response genes, and we also show that transcriptional dysregulation of interferon response genes likewise occurs in primary cord blood–derived MKs that lack nuclear ETV6. Collectively, these data indicate that MKs of patients with *ETV6* germline mutations also exhibit this proinflammatory transcriptional signature.

*ETV6* germline variants have been shown to reduce the transcriptional repressive activity of ETV6 (10). This loss of repression can be attributed to the dominant negative effect of the cytoplasmic mislocalization of ETV6, whereby WT ETV6 dimerizes with mutant copies and is removed from the nucleus (11). However, *ETV6* variants have been shown to mislocalize to the cytoplasm in different degrees (11, 15). We confirmed this with our findings in patient-derived PBMCs, in which ETV6 R369Q was observed to have a lesser degree of cytoplasmic localization compared with ETV6 P214L, suggesting other potential mechanisms by which transcription is derepressed. Despite these findings, total protein levels of ETV6 in P214L patient samples are equivalent to those in healthy controls (11). Indeed, some *ETV6* variants have been also shown to reduce homeostatic transcriptional repression through decreased DNA-binding capacity (12, 15). Our findings of increased interferon response gene expression in cells lacking nuclear ETV6 (both primary PBMCs and primary cord blood–derived MKs) suggest that nuclear ETV6 is required for HDAC3-mediated repression of these key interferon response targets. Our conclusions are supported by previous studies examining ETV6-binding sites, in which IRF motifs were highly represented in regions that were bound by ETV6 (32). Overall, our results indicate that genetic disruption of *ETV6* results in deregulation of interferon response genes, perhaps generating a proinflammatory bone marrow environment that affects megakaryopoiesis and overall hematopoiesis, predisposing to the development of myelodysplasia. Interestingly, an inflammatory bone marrow environment and predisposition to myelodysplasia is also observed in patients with RUNX1 mutations (33), another key ETS transcription factor involved in megakaryopoiesis. Aberrant innate immune activation plays a central role in the pathogenesis of myelodysplastic syndrome (34), and gene expression profiles of myelodysplastic CD34^+^ cells demonstrate upregulation of interferon response genes (35). Altogether, this suggests that ETS dysfunction could play a role in the pathogenesis of myelodysplasia. Ongoing studies in our laboratory are testing the hypothesis that MKs are critical for maintaining bone marrow homeostasis, perhaps acting as “gatekeepers” from dysregulated inflammation and subsequent myelodysplasia.

HDAC activity has been associated with normal MK maturation and platelet production. It has been proven critical for mass production of platelets in vitro (36), and HDAC inhibitors (HDACi), widely used to treat malignancies, have been associated with thrombocytopenia, likely due to decreased megakaryopoiesis and thrombopoiesis (37). The mechanism for HDACi altering platelet production is poorly understood, but some have suggested either direct toxic effects on hematopoietic progenitor cells and late-stage MKs (37) or the inhibition of proplatelet formation through increasing levels of acetylated tubulin, subsequently altering the microtubule dynamics and organization required for proper proplatelet formation (38). Of note, HDACi-treated MKs downregulate the expression of notable hematopoietic transcription factors, including GATA-1, NF-E2, and FLI1, among others (39). Other MK-specific studies demonstrate that treatment with HDACi increased the number of MK/erythroid progenitors, with an accompanied inhibition of MK differentiation (40), which is similar to our observation that patients with *ETV6* germline mutations showed small, immature, hypolobulated MKs (11) and increased progenitors in peripheral blood. Most importantly to our findings, pan-HDAC inhibition in primary MKs is associated with decreased proplatelet formation (37). In our study, we showed that cord blood–derived MKs lacking ETV6 exhibit decreased proplatelet formation, which is also observed in CD34^+^ derived MKs from ETV6 P214L patients (12) and cord blood–derived MKs treated with HDACi (37), suggesting that ETV6 and HDAC3 in combination regulate key promoters required for proplatelet formation. These key targets are likely enriched in cytoskeletal biology, as proplatelet formation has been shown to be dependent on structural rearrangement (41–43).

In summary, our genetic and molecular studies in patient-derived PBMCs demonstrated a potential mechanism for ETV6-mediated thrombocytopenia and predisposition to hematopoietic malignancies. Our findings suggest that ETV6 is required for HDAC-mediated effects on MK differentiation, and the exclusion of ETV6 shifts the balance of histone and protein acetylation, resulting in defective proplatelet formation and clinical thrombocytopenia. These findings may have implications for the effects of HDACi on patient platelet counts. Additionally, we have identified a subset of differentially expressed mRNAs that encompass a large number of interferon response genes, suggesting that ETV6 is relevant not only for MK development, but also bone marrow homeostasis.

## Methods

### Immunofluorescence.

All ETV6 P214L patients in the cohort were recruited at the Hematology Clinic at Children’s Hospital of Michigan. All ETV6 R369Q patients in the cohort were recruited at the Hematology Clinic at Children’s Mercy Hospital. 100,000 PBMCs were resuspended in buffer (1× PBS [Gibco] supplemented with 5% FBS) and spun down on coated slides (Shandon Double Cytoslide, Thermo Fisher Scientific) using Shandon Cytospin (Thermo Fisher Scientific) at 350*g* for 5 minutes. Cells were fixed in 4% paraformaldehyde for 15 minutes before blocking (5% FBS) and permeabilization (0.3% Triton X-100) for 1 hour at room temperature. Staining was directed against ETV6 (Sigma Prestige), HDAC3 (Cell Signaling Technologies), Sin3A (Cell Signaling Technologies), NCOR1 (Cell Signaling Technologies), and NCOR2 (Abcam) overnight at 4°C. Cells were washed 3 times with 1× PBS (Gibco) before incubation with anti-rabbit Alexa Fluor 568 (Life Technologies) for 1 hour at room temperature. Cells were counterstained with DAPI, 1 μg/μL (Thermo Fisher Scientific), for 10 minutes at room temperature. Slides were mounted with ProLong Antifade mounting media (Thermo Fisher Scientific). Staining specificity was determined by omission of primary antibody. Immunofluorescence image were acquired at ×60, plan apochromat (NA) 1.4 (Olympus FV1000), and analyzed (FV-Viewer Software, FV10-ASW, version 4.2b). Quantification of nuclear and cytoplasmic intensity was measured using the ImageJ macro (NIH) (http://dev.mri.cnrs.fr/attachments/download/1931/Intensity_Ratio_Nuclei_Cytoplasm.ijm). Images were exported to ImageJ (NIH) for final presentation.

### Immunoprecipitation.

LRS chambers were obtained from consented regular blood donors at Children’s Hospital Colorado, Aurora, Colorado, USA. PBMCs were lysed and protein complexes were purified by centrifugation. Protein complexes were incubated with rec-Protein G-sepharose beads (Invitrogen) and antibodies directed against ETV6 (Sigma Prestige) overnight at 4°C on a rocking platform. After incubation, beads were sequentially washed 3 times with 1× PBS (Gibco). Beads were boiled for 10 minutes in 2× SDS-PAGE sample buffer (Bio-Rad) to elute pulldown products for Western blot analysis. Protein eluate was run on Tris-Glycine SDS-page gel (Bio-Rad) and transferred to nitrocellulose membranes. Membranes were blocked in tris-buffered saline with 0.1% Tween-20 containing 5% milk at 4°C for 1 hour and were probed with HDAC3 (Cell Signaling Technologies), NCOR2 (Abcam), and α-tubulin (Abcam) primary antibodies on a shaker at 4°C overnight, followed by 1 hour of room temperature incubation with HRP-conjugated secondary antibodies (Invitrogen). Chemoluminescence was recorded using the automated Gel Doc XR+ system (Bio-Rad).

### PBMC isolation and culture.

LRS chambers were obtained from consented regular blood donors at Children’s Hospital Colorado. Whole blood was subject to Ficoll-Paque (GE Healthcare) separation for PBMC isolation following manufacturer guidelines. Mononuclear cells were counted using Trypan blue exclusion and resuspended (1 × 10^6^/mL) and cultured in RPMI 1640 media (Gibco) supplemented with 10% FBS (Thermo Fisher Scientific) and 1% Penicillin-Streptomycin (Gibco) and were maintained at 37°C in 5% CO_2_ for 24 hours before electroporation.

### siRNA knockdown.

Cultured PBMCs were subject to siRNA knockdown using 50 nM pooled siRNA against ETV6 (Dharmacon). Electroporation of siRNAs was performed using the Neon Transfection system (Thermo Fisher Scientific). All electroporation conditions were carried out according to the manufacturer’s guidelines (2150 V, 20 ms, 1 pulse). Total protein lysates were collected 48 hours after electroporation and resolved on Tris-Glycine SDS-PAGE gels as above. Membranes were probed with antibodies against anti-acetylated histone H3 (Cell Signaling Technologies) and total histone H3 (Cell Signaling Technologies).

### CD34^+^ isolation and culture.

Deidentified umbilical cord blood units were obtained from ClinImmune, Aurora, Colorado, USA. Mononuclear cells were isolated by density gradient centrifugation using Ficoll-Paque Plus (GE Healthcare). CD34^+^ cells were affinity purified from using the CD34 Microbead Kit (Miltenyi Biotech), resuspended (3 × 10^4^ cells/mL), and cultured in serum-free media containing IMDM (Gibco) supplemented with 20% BIT 9500 (Stemcell Technologies), 10 μg/mL LDL (Calbiochem), 55 μM 2-Mercaptoethanol (Gibco), 1% Penicillin-Streptomycin (Gibco), 2 mM L-glutamine (Gibco), and 50 ng/mL thrombopoietin (R&D Biosystems).

### CD34^+^ CRISPR deletion.

48 hours after isolation, CD34^+^ cells were electroporated using the Neon transfection system (Thermo Fisher Scientific) with single guide RNA (sgRNA) directed against ETV6 and Cas9 plasmid (Synthego) as previously described (44). Briefly, 1 μg sgRNA and 1 μg Cas9 were complexed for 30 minutes at room temperature. The RNP complex electroporated into cells using optimized conditions (1600 V, 10 ms, 3 pulses) (44). Total protein lysates were collected 48 hours after electroporation and resolved on Tris-Glycine SDS-PAGE gels (Bio-Rad) as above. Membranes were probed for acetylated histone H3 and total histone H3 as above.

### Single-cell RNA-Seq.

All ETV6 P214L patients in the cohort were recruited at the Hematology Clinic at Children’s Hospital of Michigan. All ETV6 R369Q patients in the cohort were recruited at the Hematology Clinic at Children’s Mercy Hospital. Isolated PBMCs were resuspended in 1× PBS (Gibco) with 0.04% weight/volume BSA. Chromium Single-cell 3′ Reagent (V2 chemistry) was used for barcoding and cDNA library preparation as per the manufacturer’s guidelines, and cDNA was sequenced using the 10× Genomics Single-cell RNA-Seq platform at the University of Colorado Genomics Core Facility (BioProject PRJNA657295).

### Analysis of single-cell-Seq.

Single-cell RNA-Seq data were initially processed by the Cell Ranger pipeline (v2.1.0) from 10× Genomics using the human reference genome (GrCH38). The generated count matrices were then analyzed using the Seurat package in R (v3). Read counts were normalized to library size, scaled by 10,000, log transformed, and filtered based on the following criteria: cells with less than 250 genes detected, proportion of the UMIs mapped to mitochondrial genes over 0.1, or high HBB gene expression were excluded from analysis; genes detected in less than 10 cells were also excluded. Cell-cell variation in gene expression driven by the number of detected molecules and mitochondrial gene expression were regressed out using linear regression. Dimensionality reduction, PCA, UMAP projection, and graph-based clustering analysis were then performed. Marker genes for each cluster were identified by Wilcoxon rank sum test comparing genes expressed in each cluster to all other clusters.

### Platelet RNA-Seq.

All ETV6 P214L patients in the cohort were recruited at the Hematology Clinic at Children’s Hospital of Michigan. All ETV6 R369Q patients in the cohort were recruited at the Hematology Clinic at Children’s Mercy Hospital. Total RNA was isolated from leukoreduced platelet preparations and stored in Trizol (Invitrogen) as previously described (45). RNA-seq libraries were prepared and bar-coded using TruSeq V2 with oligo selection (Illumina). 50 cycle single-end reads were generated on a single lane of the HiSeq 2000 (Illumina) and aligned using Novoalign (Novocraft Technologies) to UCSC genome version hg19 with known and shuffled splice junctions. Assignment of read counts to transcripts was performed using the USeq analysis suite. Normalization of read counts and differential expression analysis was performed with DESeq2 (46). Sample-to-sample variability in the level of leukocyte transcripts, which can significantly alter read counts, was corrected for by including the ratio of PTPRC (leukocyte marker) and CD45/ITGA2B (platelet marker) as a factor in the model for significance testing (BioProject PRJNA656812).

### PBMC RNA-Seq.

Total RNA was isolated using the RNeasy RNA extraction kit (QIAGEN) as per the manufacturer’s guidelines. RNA purity and concentration were measured on a NanoDrop (Thermo Fisher Scientific). Nugen Universal Plus mRNA-seq Library Preparation (Tecan) was used for next-generation library construction per the manufacturer’s protocols. The directional mRNA template libraries were then sequenced as paired-end 150 bp reads on the Novaseq 6000 platform at the University of Colorado Genomics Core Facility. Derived sequences were analyzed by applying a custom computational pipeline consisting of the open-source gSNAP, Cufflinks, and R for sequence alignment and ascertainment of differential gene expression (47). In short, reads generated were mapped to the human genome (GrCh38) by gSNAP (48), expression (FPKM) was derived by Cufflinks (49), and differential expression was analyzed with ANOVA in R. Genes significant at an FDR < 0.05 were submitted to pathway analysis using Ingenuity Pathway Analysis (QIAGEN) to identify pathways of interest that were modified by ETV6 knockdown (BioProject PRJNA657293).

### Immunophenotyping.

Isolated PBMCs were examined for cell surface expression by flow cytometry. Expression of surface markers was determined using the following antibodies: CD34 BV421 (BD, clone 581), CD10 APC (BioLegend, clone HI10a), CD123 BV711 (BD, clone 7G3), CD3 FITC (BioLegend, clone HIT3a), CD90 PE (BD, clone 5E10), CD38 CF594 (BD, clone HIT2), CD19 PECy7 (BD, clone HIB19), CD20 BV605 (BD, clone 2H7), and CD45RA A700 (BD, clone HI100). Cells were stained for 30 minutes at 4°C. Single-color controls were obtained using CompBead Anti-Mouse Ig, k/Negative Control Compensation Particles (BD Biosciences). Data were acquired on BD FACSAria Cell Sorter and analyzed with FlowJo software (BD Biosciences).

### Plasma cytokine analysis.

All ETV6 P214L patients in the cohort were recruited at the Hematology Clinic at Children’s Hospital of Michigan. All ETV6 R369Q patients in the cohort were recruited at the Hematology Clinic at Children’s Mercy Hospital. Whole blood was collected into EDTA-treated tubes (BD Biosciences). Cells and platelets were removed by centrifugation for 15 minutes at 2000*g* at 4°C. 500 μl aliquots of plasma were maintained at –80°C. The V-PLEX Human cytokine 30-Plex kit (Mesoscale Discovery) was used to detect Eotaxin, Eotaxin-3, GM-CSF, interferon-γ, IL-1α, IL-1β, IL-2, IL-4, IL-5, IL-6, IL-7, IL-8, IL-8 (HA), IL-10, IL-12/IL-23p40, IL-12p70, IL-13, IL-15, IL-16, IL-17A, IP-10, MCP-1, MCP-4, MDC, MIP-1α, MIP-1β, TARC, TNF-α, TNF-β, and VEGF-A as per the manufacturer’s guidelines. Plates were read on QuickPlex SQ120 (Mesoscale Discovery).

### MK differentiation and proplatelet formation.

CD34^+^ cord blood–derived cells were maintained in serum-free media as described above and were analyzed for proplatelet formation 12–14 days after electroporation as previously described (50). 100,000 cells were plated per replicate, cells demonstrating proplatetets were counted (×20 magnification, light microscope) and quantified relative to total cells using Trypan blue exclusion.

### RNA-Seq and analysis.

Samples were prepared according to library kit manufacturer’s protocol (Illumina), indexed, pooled, and sequenced on an Illumina NovaSeq. Base calls and demultiplexing were performed with Illumina’s RTA version 1.9 and bcl2fastq2 software with a maximum of 1 mismatch in the indexing read. RNA-seq reads were then aligned to the Ensembl release 76 primary assembly with STAR version 2.5.1a (51). Gene counts were derived from the number of uniquely aligned unambiguous reads by Subread:featureCount version 1.4.6-p5 (52). Isoform expression of known Ensembl transcripts was estimated with Salmon version 0.8.2 (53). Sequencing performance was assessed for the total number of aligned reads, total number of uniquely aligned reads, and features detected. The ribosomal fraction, known junction saturation, and read distribution over known gene models were quantified with RSeQC version 2.6.2 (54).

All gene counts were then imported into the R/Bioconductor package EdgeR (55), and TMM normalization size factors were calculated to adjust for samples for differences in library size. Ribosomal genes and genes not expressed in the group with the smallest size minus 1 sample greater than 1 count per million were excluded from further analysis. The TMM size factors and the matrix of counts were then imported into the R/Bioconductor package Limma (56). Weighted likelihoods based on the observed mean-variance relationship of every gene and sample were then calculated for all samples with the voomWithQualityWeights (57). The performance of all genes was assessed with plots of the residual SD of every gene to their average log count with a robustly fitted trend line of the residuals. Differential expression analysis was then performed to analyze for differences between conditions, and the results were filtered for only those genes with Benjamini-Hochberg FDR-adjusted *P* values less than or equal to 0.05.

For each contrast extracted with Limma, global perturbations in known Gene ontology (GO) terms, MSigDb, and KEGG pathways were detected using the R/Bioconductor package GAGE (58) to test for changes in expression of the reported log_2_ fold changes reported by Limma in each term versus the background log_2_ fold changes of all genes found outside the respective term. The R/Bioconductor package heatmap3 (59) was used to display heatmaps across groups of samples for each GO or MSigDb term with a Benjamini-Hochberg FDR-adjusted *P* value of less than or equal to 0.05. Perturbed KEGG pathways where the observed log_2_ fold changes of genes within the term were significantly perturbed in a single direction versus background or in any direction compared with other genes within a given term, with *P* values less than or equal to 0.05 were rendered as annotated KEGG graphs with the R/Bioconductor package Pathview (60) (BioProject PRJNA657292).

### Data display.

Data visualizations were created in GraphPad Prism 8 and Adobe Illustrator (version CC 2020). Data are presented as the mean ± SD.

### Statistics.

Analyses were performed using GraphPad Prism 8. Results were considered statistically significant if the *P* value was less than 0.05. Normality of the data were assessed using the Anderson-Darling test or Shapiro-Wilk test in cases of smaller sample size. For tests between 2 groups of normal data, an unpaired *t* test was used. Mann-Whitney *U* test was used for data where sample size was too small to assess normality. All statistical tests were 2 tailed. Experimental numbers are reported as *n* = *x*.

### Study approval.

All ETV6 P214L patients in the cohort were recruited at the Hematology Clinic at Children’s Hospital of Michigan. All ETV6 R369Q patients in the cohort were recruited at the Hematology Clinic at Children’s Mercy Hospital. The study received Institutional Review Board approval from the University of Colorado Anschutz Medical Campus. Deidentified umbilical cord blood units were obtained from ClinImmune under protocols approved by the institutional review board of the University of Colorado. LRS chambers from donors at Children’s Hospital Colorado were obtained under methods approved by the institutional review board of the University of Colorado. Written informed consent was obtained for all participants. Studies were performed in accordance with the Declaration of Helsinki.

## Author contributions

MHF and JDP devised the research strategy. MHF, GDK, BS, and CJ designed experimental methodologies. MHF, GDK, and KJ performed sequencing analyses. MC, MR, JF, and MAC contributed vital reagents, samples, and clinical information. CCP, AGH, CJ, EMP, and JR contributed with analysis of data and reagents. MHF and JDP wrote the manuscript. All authors edited the manuscript and approved its final version.

## Supplementary Material

Supplemental data

## Version 1 08/25/2020

In-Press Preview

## Version 2 09/17/2020

Electronic publication
