# Supplementary material for: ETV6 germline mutations cause HDAC3/NCOR2 mislocalization and upregulation of interferon response genes
Source: JCI Insight. 2020 Sep 17;5(18):e140332. doi: 10.1172/jci.insight.140332 (PMC7526537; doi:10.1172/jci.insight.140332)
Supplement: Supplemental data [file jciinsight-5-140332-s211.pdf]

Figure S1

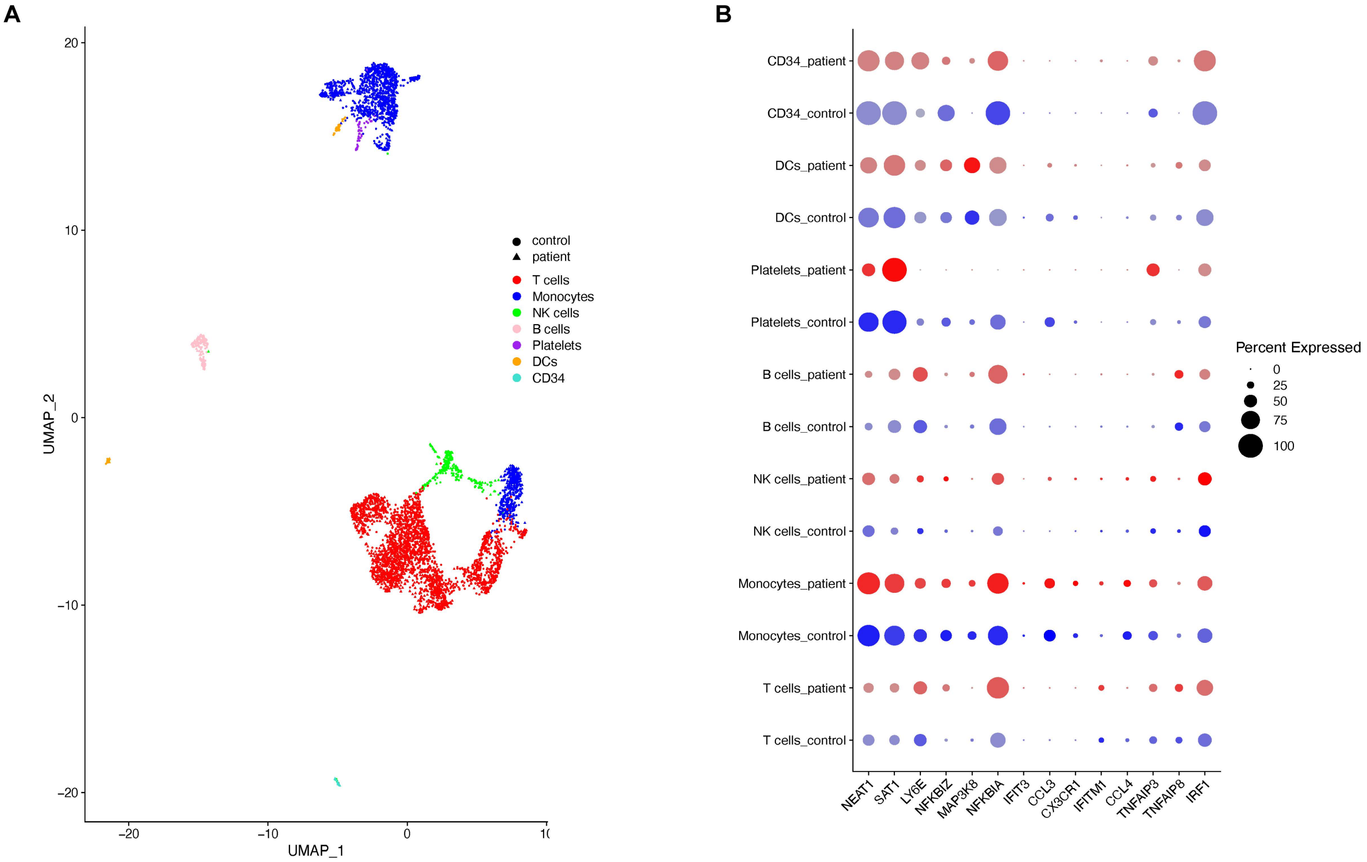

Figure S1. Cytoplasmic localization of ETV6 R369Q patients' PBMCs drives upregulation of interferon response genes
(A) UMAP plot of cells sequenced from healthy controls (circle) and ETV6 R369Q patients (triangle). **(B)** Dot plot of top differentially expressed interferon response genes split by ETV6 R369Q patients versus age and gender matched controls.

Figure S2

Master Regulator	Molecular Function	p value	z score
HDAC3	Transcriptional regulator	2.88E-15	3.051
IFNG	Cytokine	1.66E-14	-3.051
TLR3	Transmembrane protein	4.70E-14	-3
TLR9	Transmembrane protein	1.62E-13	-3
NCR3	Transmembrane protein	3.71E-13	0
IFNA4	Cytokine	4.87E-13	-1.941
RLN2	Other	5.07E-13	-0.577
CSF2RB	Transmembrane protein	5.42E-13	-0.577
TNFRSF8	Transmembrane protein	7.01E-13	-3.317
NOTCH2	Transmembrane protein	9.81E-13	-0.302

Figure S2. Ingenuity Pathway Analysis Upstream Regulators
Ingenuity Pathway Analysis predicted HDAC3 to be the upstream regulator of the upregulated proinflammatory interferon response genes observed in the ETV6 P214L and R369Q PBMCs.

Figure S3

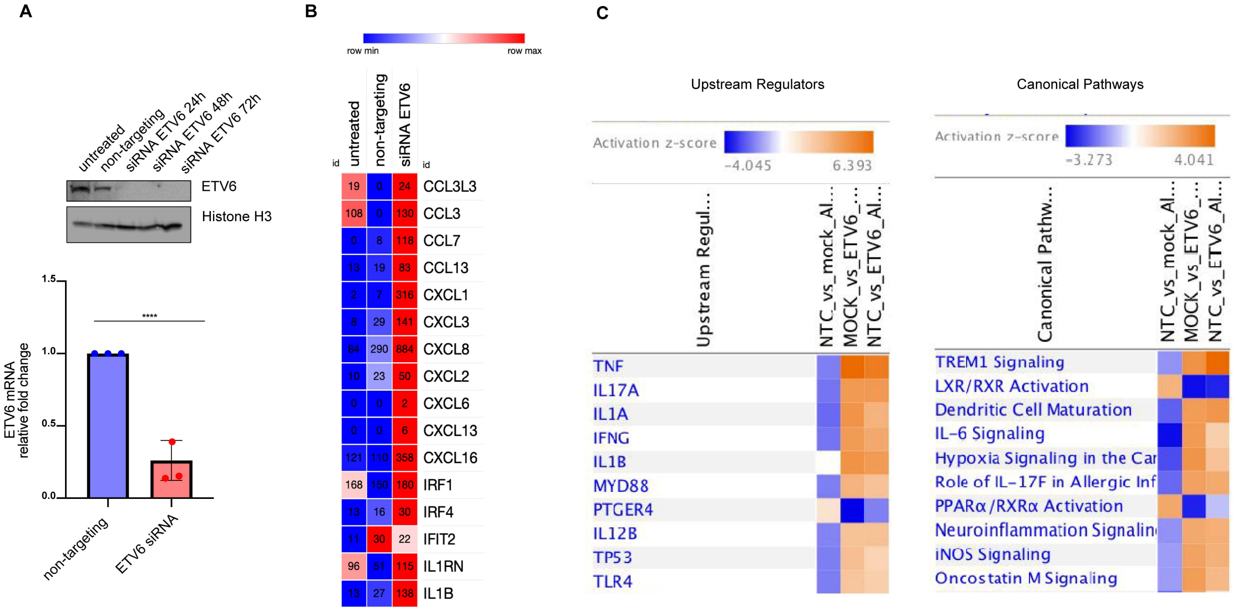

Figure S3. Knockdown of ETV6 in PMBCs results in a proinflammatory transcriptional signature
(A) Western blot demonstrating protein knockdown of ETV6 upon targeted siNRA knockdown in healthy PBMCs. Representative image of two independent experiments. mRNA knockdown of ETV6 by qPCR, relative to GAPDH. ETV6 mRNA levels significantly decrease with ETV6 targeted siRNA in healthy PBMCs (Mann Whitney U test, $p<0.0001$, three independent experiments). **(B)** Proinflammatory interferon response genes increase in ETV6 knockdown compared to non-targeting control siRNA. Analysis of three independent experiments, results not statistically significant due to variability in ETV6 knockdown efficiency. **(C)** Ingenuity PathwayAnalysis identifies monocyte and macrophage activation upon ETV6 knockdown in PBMCs.

Figure S4

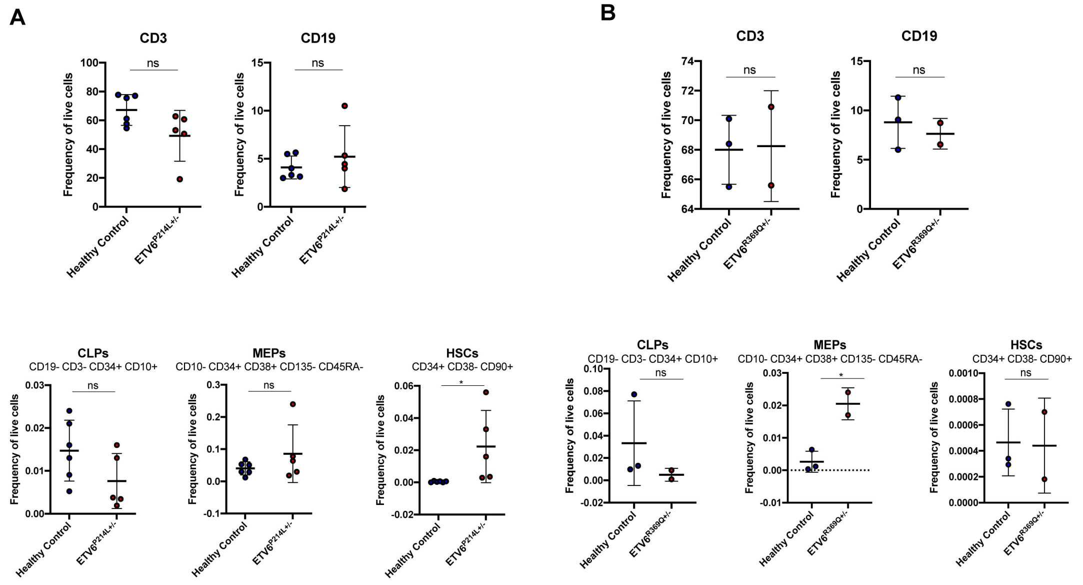

Figure S4. Immunophenotyping of ETV6 P214L and R369Q

(A) Flow cytometric analysis of PBMCs from healthy controls and ETV6 P214L patients. Frequencies of CD3 T cells, CD19 B cells, CLPs (CD19-, CD3-, CD34+, CD10 +), and MEPs (CD10- CD34+ CD38+ CD135-CD45RA-) were not significantly different between patients and controls, while the frequency of HSCs (CD34+, CD38-, CD90+) were significantly increased (unpaired t-test, p=0.0393) in affected patients. **(B)** Flow cytometric analysis of PBMCs from healthy controls and ETV6 R369Q patients. Frequencies of CD3 T cells, CD19 B cells, CLPs (CD19-, CD3-, CD34+, CD10+), and HSCs (CD34+, CD38-, CD90+) were not significantly different between patients and controls, while the frequency of MEPs (CD10- CD34+ CD38+ CD135- CD45RA-) were significantly increased (unpaired t-test, p=0.0152) in affected patients.

Figure S5

	Healthy Controls	ETV6 P214L
Eotaxin	84.75	301.2
Eotaxin-3	8.875	8.197
GM-CSF	0.2339	0.1875
IFN-γ	3.18	2.057
IL-10	0.2952	0.499
IL-12/23p40	175.5	103.1
IL-12p70	0.9685	0.1774
IL-13	1.099	0.8881
IL-15	1.635	1.69
IL-16	234.4	264.8
IL-17A	3.376	1.714
IL-1α	0.9152	1.206
IL-1β	0.3666	0.0537
IL-2	0.1373	0.2791
IL-4	0.02356	0.02432
IL-5	0.4194	0.5192
IL-6	0.7156	0.4144
IL-7	1.297	0.6616
IL-8	2.131	2.818
IP-10	326.1	358.3
MCP-1	71.39	69.47
MCP-4	26.66	76.27
MDC	836.3	682.9
MIP-1α	11.58	13.72
MIP-1β	55.51	76.94
TARC	24.09	27.02
TNF-α	2.086	2.018
TNF-β	0.2974	0.1892
VEGF	11.48	23.12

Figure S5. Cytokine analysis of ETV6 P214L serum
29 cytokines, chemokines, and proinflammatory molecules were analyzed in the serum of healthy controls (n=4) and patients carrying ETV6 P214L (n=4) using a MesoScale Discovery multiplex assay. Mean values of 4 samples with technical duplicates. No significant differences were observed between healthy controls and patients.

Figure S6

DAVID Cluster	Enrichment Score	Genes
**Extracellular Matrix and Cell Adhesion**	1.8	ADAM metallopeptidase domain 12 (ADAM12)
		EGF containing fibulin like extracellular matrix protein 2 (EFEMP2)
		Wnt family member 11 (WNT11)
		caspase 9 (CASP9)
		catenin delta 2 (CTNND2)
		clusterin (CLU)
		erythropoietin receptor (EPOR)
		extracellular matrix protein 1 (ECM1)
		filamin A (FLNA)
		intercellular adhesion molecule 5 (ICAM5)
		laminin subunit beta 2 (LAMB2)
		laminin subunit gamma 1 (LAMC1)
		matrix metallopeptidase 1 (MMP1)
		myosin light chain 9 (MYL9)
		protein tyrosine phosphatase, receptor type F (PTPRF)
		syndecan 4 (SDC4)
		talin 1 (TLN1)
		tenascin XB (TNXB)
		Von Willebrand factor (VWF)
**Cytoskeleton**	1.78	PDZ and LIM domain 7 (PDLIM7)
		WAS protein family member 3 (WASF3)
		dynamin 1 (DNM1)
		dynein axonemal heavy chain 2 (DNAH2)
		dynein axonemal light chain 4 (DNAL4)
		dynein cytoplasmic 1 intermediate chain 1 (DYNC1I1)
		echinoderm microtubule associated protein like 6 (EML6)
		filamin A (FLNA)
		golgin A2 (GOLGA2)
		microtubule associated protein 1A (MAP1A)
		myosin XVIIIB (MYO18B)
		myosin light chain 9 (MYL9)
		septin 4 (SEPT4)
		talin 1 (TLN1)
		villin 1(VIL1)

Figure S6. DAVID Functional Annotation Clustering analysis of ETV6 P214L platelet transcripts 141 genes were significantly downregulated in platelets derived from patients with the P214L mutation (n=2) compared to all controls (n=5). DAVID analysis of transcripts significantly downregulated in patients with ETV6 P214L designated two functional clusters with enrichment scores greater than or equal to 1.3.
